# The Impact of Financial Development on Renewable Energy Consumption: A Multidimensional Analysis Based on Global Panel Data

**DOI:** 10.3390/ijerph20043124

**Published:** 2023-02-10

**Authors:** Zhongye Sun, Xin Zhang, Yifei Gao

**Affiliations:** 1 School of Economics and Trade, Henan University of Technology, Zhengzhou 450001, China; 2 School of Law, Zhejiang Gongshang University, Hangzhou 310018, China

**Keywords:** financial development, renewable energy consumption, national heterogeneity, dynamic panel model, system GMM

## Abstract

In this paper, we examined the impact of financial development on renewable energy consumption from a global perspective based on a dynamic panel model and panel data of 103 economies. We conducted the research from the different levels of financial development using an index system including nine variables, and also explored national heterogeneity by dividing samples into developed economies and developing economies. The empirical results indicated that the financial development had a positive impact on renewable energy consumption from the macro perspective, and this effect was mainly driven by the development of a financial institution (mainly including bank). Further analysis on the depth, access, and efficiency of a financial institution and financial market (mainly including stock market and bond market) revealed that all three aspects of a financial institution had a positive influence on renewable energy consumption, while this effect only existed in the aspect of efficiency for a financial market. The investigation of national heterogeneity showed that the financial development performed well in promoting renewable energy consumption in developed economies, while this positive effect only existed for financial institutions in developing economies. We suggest to policymakers to attach importance to the positive effect of financial development when formulating renewable-energy-related policies, and provide a system guarantee for renewable energy enterprises concerning financial sectors in developing economies.

## 1. Introduction

Energy is one of the fundamental elements to maintain social operation. It not only relates to the daily life of residents, but also is an important driving force for the development of an economy. Therefore, policymakers regard energy supply as an important topic. Due to the rapid growth of the population and economy, energy consumption dramatically increased in recent years. Up to now, traditional fossil fuels, such as oil and coal, are still the main source of energy consumption. On the one hand, the extensive use of traditional fossil fuels has dramatically increased carbon emissions, which has caused a series of environmental problems. On the other hand, as nonrenewable energies, traditional fossil fuels are limited; therefore, the ever-increasing energy demand may lead to serious energy shortage. Especially for energy-deficient countries, energy supply cannot be guaranteed in the long term, which will remarkably impede economic growth and even affect social stability. Under these circumstances, how to ensure the stability and sustainability of energy supply has become an important issue faced by most economies around the world.

To cope with this outstanding issue, most scholars conducted their studies from the perspective of “amount”, namely, hunting for those factors that may influence energy consumption, thereby formulating corresponding energy policies to restrain energy use or improve energy efficiency. Many factors have been proposed that can affect energy consumption in recent years, such as industrial structure, trade openness, and foreign direct investment.

Besides all these factors, financial development has also been deemed a non-negligible element that can influence energy consumption, and a series of literature analyzed the impact of financial development on energy consumption from both theoretical and empirical perspectives [1,2,3].

In recent years, scholars concentrating on the realm of energy and environment realized that another effective way to cope with environmental pollution and the potential energy shortage was to exploit and apply renewable energy. Compared with traditional fossil energy, renewable energy dramatically reduces carbon emissions, which could effectively restrain the greenhouse effect and improve environmental quality; in addition, renewable energy is theoretically inexhaustible, which can provide sustainable power for social operation. Actually, with the progress of related technologies, the utilization cost of renewable energy will be significantly reduced; furthermore, limited by geographical factors, traditional fossil fuels are unevenly distributed in the world, and most reserves are concentrated in a few countries. This leads to a potential risk of energy supply and a large fluctuation of the international energy price. By comparison, renewable energy appears to be of various forms, which are generated from different sources, such as solar, wind, and geothermal energy. All these forms could largely overcome geographical limitations, which fossil fuels have. Most countries can develop renewable energy by relying on their own advantages, therefore alleviating energy supply problems.

The most prominent factor that promotes the development of a renewable energy industry is financial support. As an emerging industry, the renewable energy industry is at the initial stage of development, which requires abundant investment in infrastructure, equipment, and personnel training. Meanwhile, the related technologies of renewable energy need to be constantly upgraded to lower production costs. Although the renewable energy industry generally obtains funding support from a government at the early stage, it needs more financing channels in its development process as policy subsidy is limited and unsustainable.

The main objective of this paper is to analyze the impact of financial development on renewable energy consumption from a multidimensional perspective based on global panel data to make a certain marginal contribution to empirical research in this field. Specifically, the main contributions and innovations are as follows: (1) Different from most literature, we analyzed the impact of financial development on renewable energy consumption from a global view, as we collected panel data of over 100 economies. It is necessary and valuable to investigate the specific countries or regions, as it can provide particular experience for energy policymaking. Likewise, it is also useful to investigate this issue from a macro perspective and temporarily leave out the specific characteristics of certain economies, as it can provide us an intuitive perception of the comprehensive effect of financial development on renewable energy consumption. (2) We attached importance to the multiple levels of financial development. Financial development refers to the level of development of a financial sector in a certain economy or region. As an abstract concept, it can be deemed and analyzed from an overall perspective or investigated from its components, such as financial institution or market. Scholars also have developed several indicators measuring the overall financial development or its components to conduct an empirical analysis. Considering the rich connotation of financial development, we regard it as a system and examine its influence on renewable energy consumption with a set of indicators in a unified framework to comprehensively explore the nexus of financial development and renewable energy consumption. (3) We investigated the heterogenous impact of financial development on renewable energy consumption between different types of economies. The characteristics of specific economies have to be neglected in the research of macro effect using a worldwide transnational sample. However, economies at the same stage of development may appear to have homogeneity features; therefore, we conducted subsample regressions by dividing samples into developed economies and developing economies to examine the national heterogeneity of the impact of financial development on renewable energy consumption.

The rest of this paper is arranged as follows: Section 2 is a literature review, which summarizes relevant theories and empirical research. Section 3 introduces the empirical strategy and data sources we adopted. Section 4 outlines the empirical results and discussions. Section 5 presents the robustness tests. Section 6 provides the conclusions and policy implications.

## 2. Literature Review

Although many economies around the world have achieved rapid economic growth and technological progress in recent decades, a series of outstanding issues, such as global warming and energy shortage, have also attracted widespread attention. Scholars have conducted research to seek sustainable development from the theoretical perspective. One of the eminent theories concerning an economy–environment nexus is the environmental Kuznets curve (EKC) hypothesis proposed by Grossman and Krueger [4]. This hypothesis indicates that economic development and environmental quality have an inverted U-shaped relationship. Specifically, at the early stage of economic development, economic growth relies on a large amount of resource input, which increases environmental pollution. However, with the further development of the economy and the progress of technology, the utilization efficiency of resources can be significantly improved, which mitigates environmental pollution. The subsequent research analyzed the relationship among environmental protection, energy consumption, and economic growth from various perspectives.

One prominent research topic is the finance–energy nexus. Sadorsky [5] pointed out that financial development could promote energy consumption by three channels. First, a developed financial system could enable consumers to obtain credit with a much lower cost, which stimulates them to purchase commodities such as automobiles, refrigerators, and washing machines, therefore increasing the overall energy demand of an economy. Second, a well-functioning financial system can also provide loans for enterprises by various financing channels with low interest rates, which encourage them to expand production scales and produce more commodities, therefore indirectly promote energy consumption. Third, from a macro perspective, as an important component of financial development, the stock market is commonly regarded as a barometer of economic conditions. An active stock market generally strengthens the confidence of investors and consumers, which further promotes economic activity and energy consumption. Conversely, other scholars [6,7,8] proposed different theoretical standpoints, that financial development could reduce energy consumption. They pointed out that enterprises tend to improve energy efficiency or reduce energy consumption in order to save the cost of production. Financial institutions and markets could provide funds for enterprises to effectively relieve their financial constraints, therefore enabling them to update production equipment and invest in energy-saving technology, consequently decreasing energy consumption. Besides, all listed companies are subject to the supervision of the public due to the requirement of regular information disclosure. To maintain fine corporate images, enterprises attempt to adopt energy conservation technologies and measures through company operations, to assume the social responsibility of environmental protection, which facilitates the goal of energy saving.

A series of literature analyzed the relationship of financial development and energy consumption from the empirical perspective. Several scholars [9,10,11] discovered that financial development has a positive influence on energy consumption, and others [12,13,14] found evidence that financial development could reduce energy consumption. Besides, the empirical results of some literature indicated that the relationship between financial development and energy consumption is complex. For instance, using panel data of 21 transitional countries and the panel smooth transition regression (PSTR) model, Yue et al. [15] discovered that the direct influence of financial development on energy consumption is insignificant, whereas the development of financial intermediation, the stock market, and financial openness have a significant effect on energy consumption in all countries or certain countries. Based on a global sample of 112 countries and dynamic two-step system GMM (generalized method of moments) estimations, Nguyen [16] found that the influence of financial development on energy consumption is positive while the institutional environment of a country is weak, and is negative while it is well developed. In addition, a few scholars [17,18] demonstrated that the effect of financial development on energy consumption is insignificant. Although research concerning the effect of financial development on energy consumption has not reached a consistent conclusion, scholars have proposed many feasible policy suggestions to cope with the potential issues of energy supply.

Due to the obvious advantages of renewable energy, such as cleanliness, inexhaustibility, and wide distribution, scholars have started to concentrate on the influence of financial development on renewable energy consumption. Several studies proposed that financial development acts as an important driving force for the development of the renewable energy industry and the increase in renewable energy demand. They considered that a developed financial institution can offer debt financing for renewable-energy-related projects with low cost, and a well-functioning stock market can allocate capital to environmentally friendly industries, which will promote output and consumption of renewable energy [19,20,21]. This viewpoint has been supported by a series of empirical literature, which discovered that financial development has a positive influence on renewable energy consumption. Kutan et al. [22] analyzed the effect of stock market development on renewable energy consumption based on a series of robust panel econometric techniques and the cross-country annual data of Brazil, China, India, and South Africa from 1990 to 2012. The results showed that stock market development could effectively promote renewable energy consumption, while the latter helps to mitigate the growth of CO2 emissions. Ji and Zhang [23] considered that developing the renewable energy sector has strategic implications against climate changes based on time series analysis with macro-level data. They discovered that financial development contributed 42.42% to the overall variation of renewable energy consumption growth. Besides, a capital market was found to be the most important element in this process. Eren et al. [24] examined the influence of financial development on renewable energy consumption based on the annual time series data of India covering the period from 1971 to 2015. Dynamic ordinary least squares (DOLS) estimation and a Granger causality test under a vector error correction model were utilized for the empirical research, and all results indicated that financial development has a statistically significant and positive influence on renewable energy consumption. Alsagr and Hemmen [25] pointed out it that is important for the transition to renewable energy sources to realize the global environmental needs based on the two-step system GMM estimation method and cross-country panel data of large industrialized emerging economies from 1996 to 2015. Their empirical investigation indicated that financial development has a significant positive influence on the transition to renewable energy. Using robust standard error regression and the dynamic GMM estimation and the panel data of 69 countries of the “Belt and Road Initiative (BRI)” covering a period from 2000 to 2014, Khan et al. [26] discovered that financial development is one of the significantly positive elements to facilitate the increase in regional renewable energy consumption, which implies that in the BRI countries, financial markets should be promoted. Shahbaz et al. [27] investigated the relationship between financial development and renewable energy consumption in 34 upper-middle-income developing countries based on panel Pedroni cointegration, Kao cointegration tests, and fully modified OLS (FMOLS) approach with the panel data from 1994 to 2015. They discovered that financial development effectively promotes the demand of renewable energy consumption, which further restrains the emissions of greenhouse gases in nature, as opposed to fossil energy. Besides, other scholars also detected empirical evidence that financial development has a positive influence on renewable energy consumption [28,29].

However, it is non-negligible that different from the mature traditional energy industry, the renewable energy industry requires tremendous and long-term funds at the early stage, and the relevant technical research and development confronts great uncertainty in the process, which significantly increases the investment risk. The financial sector, especially commercial banks, is sensitive to such long-term and high-risk loans, and generally implements a strict risk control system, which may prevent it from lending to the renewable energy industry and consequently restrain the output and consumption of renewable energy. Actually, several scholars have discovered empirical evidence that financial development has no effect or a negative effect on renewable energy consumption. Assi et al. [30] examined the impact of financial development on renewable energy consumption in ASEAN +3 economies utilizing the panel ARDL (autoregressive distributed lag) analysis and the Dumitrescu–Hurlin panel causality test with the panel data from 1998 to 2018. The empirical results indicated that the influence of financial development on renewable energy consumption is insignificant. Lei et al. [31] analyzed the asymmetric effect of financial development on renewable energy consumption in China based on a nonlinear ARDL approach and time series data covering the period from 1990 to 2019. They discovered that financial development has no obvious effect on renewable energy consumption. Saadaoui [32] investigated the relationship between financial development and renewable energy consumption in the Middle Eastern and North African (MENA) region by using the ARDL pooled mean group (PMG) method and the cross-country panel data from 1990 to 2018. The empirical research showed that financial development has no significant impact on the process of energy transformation in the long run; nevertheless, such impact becomes negative and significant in the short run. Saadaoui and Chtourou [33] researched the effect of financial development on renewable energy consumption based on symmetric and asymmetric ARDL with the data from 1984 to 2017 in Tunisia. They found that financial development has a negative and significant influence on renewable energy consumption when nonlinear approaches are used. Using panel data from 1990 to 2018 in selected countries of South Asia, Bin Amin et al. [34] examined the relationship between financial development and renewable energy consumption, and found that in the long-term, financial development has a negative impact on the propensity of renewable energy consumption by 0.07%–0.15%.

Besides, several scholars explored the influence of financial development on renewable energy consumption from different perspectives, such as various aspects of financial development or moderating effect, which drew some other conclusions. Le et al. [35] investigated the relationship between financial development and the deployment of renewable energy by utilizing a two-step GMM approach and the cross-country panel data of 55 economies from 2005 to 2014. The empirical results showed that the development of the financial sector has a remarkable influence on the deployment of renewable energy; however, this effect could only be found in the group of high-income economies. It was statistically insignificant for the group of middle-income and low-income economies. Using a generalized method of moments estimation and the cross-sectionally augmented autoregressive distributed lag estimator, Saygin and Iskenderoglu [36] analyzed the influence of financial development on renewable energy consumption with the panel data of 23 developed countries from 1990 to 2015. The empirical estimation showed that when banking variables are utilized to measure financial development, the coefficients are positive and strongly significant. However, when financial development is measured by stock market variables, the coefficients are negative and statistically insignificant in all models. Based on panel threshold models and the cross-country panel data of 35 countries from 1996 to 2018, Yu et al. [37] researched the relationships among financial development, information, and communication technology (ICT) and renewable energy consumption. They found that both financial development and ICT have a positive effect on renewable energy consumption, and further analysis of the moderating effect indicated that when ICT is used as the moderating variable, the influence of financial development on renewable energy consumption presents a significant threshold effect. The positive relationship between financial development and renewable energy consumption becomes stronger when the level of ICT increases. Therefore, they suggested that governments could attach importance to the integration of financial development and ICT when formulating renewable energy policies. Raza et al. [38] examined the nonlinear relationship between financial development and renewable energy consumption based on the panel smooth transition regression (PSTR) model and the panel data of top renewable energy consumption countries from 1997 to 2017. The empirical analysis indicated that all proxy indicators of financial development had a positive effect on renewable energy consumption. Moreover, considering the moderating effects with several indicators, the industrial structure and economic growth presented a significantly positive association in both low and high regimes; nevertheless, with regard to the indicator of population, the relationship between financial development and renewable energy consumption was negative when population was in low regime, while the relationship became positive when population was in high regime. Wang and Dong [39] investigated the linear and nonlinear relationship between financial development and renewable energy consumption with a fixed-effect model and a panel threshold model based on cross-country panel data of G20 countries from 2005 to 2018. They discovered that the linear impact of financial development on renewable energy consumption is insignificant, while the nonlinear influence is significant. Financial development could effectively promote renewable energy consumption only when certain factors, such as population, affluence, and technology, exceed a certain level; otherwise, financial development would have a negative impact on renewable energy consumption. Saygin and Iskenderoglu [40] studied the influence of financial development on renewable energy consumption based on system GMM estimation and the annual frequency data of 20 emerging countries between 1990 and 2015. Their empirical analysis indicated that when utilizing banking and stock market variables as proxy indicators, the influence of financial development on renewable energy consumption is insignificant, while financial development promotes renewable energy consumption if it is measured by stock market capitalization.

Theoretical analysis has proposed several channels in which financial development can increase renewable energy consumption, which has been detected by a series of empirical work. However, we can notice that other scholars obtained opposite or different results based on their empirical analysis. This indicates that we could not consider the relationship between financial development and renewable energy consumption from a single perspective. As discussed above, the development of the renewable energy industry requires tremendous and long-term funds that contain tremendous risk, which may prevent the financial sector from lending to it. Therefore, we should attach importance to the potential adverse factors and keep an objective view when analyzing the impact of financial development on renewable energy consumption.

Although the relevant literature does not obtain a consistent conclusion, they have dramatically developed the theory of energy finance, which provides foundations and directions for the future exploration of the nexus between financial development and renewable energy consumption. Besides, the corresponding empirical evidence could facilitate financial support energy policymaking for different economies.

However, there exist several limitations in the relevant literature: First, most scholars adopted individual or regional samples as research objects, and few of them conducted their research from the worldwide perspective by using global panel data, which may provide us the empirical evidence on the macro level. Second, due to the utilization of different methods and data, it is very difficult to conduct the contrastive analysis of different studies. Third, although several scholars have attached importance to the multiple levels of financial development, many studies have adopted only a single or a few indexes to measure the level of financial development.

Therefore, in this paper, we attempted to analyze the impact of financial development on renewable energy consumption from a multidimensional perspective based on a dynamic panel model and global panel data to contribute to the empirical exploration of this topic.

## 3. Empirical Strategy and Data Sources

In this section, we introduced the methodology and data we adopted in empirical research. The main methodology we used is the dynamic panel model, which will be introduced in Section 3.1 in detail. To verify the fitness of the selected data, several pretests, including correlation coefficient test, panel unit root test, and panel cointegration test, should be conducted before model estimation. However, because the corresponding methodologies of pretests adopted in this paper are widely used in the literature, which readers are quite familiar with, following the general practice, we will briefly introduce them before model estimation in Section 4.

### 3.1. Methodology

To achieve the research objectives of this paper, we established the dynamic panel model to conduct empirical analysis. First, the panel model is more efficient for estimation than cross-sectional or time series models, and the large sample in our research further increased the accuracy of estimation results; second, compared with a traditional static panel model, a dynamic panel model includes a lag term of a dependent variable, which can enhance the credibility of the regression results by eliminating the impact of unobservable factors. It is also consistent with reality as the lag term reflects the dynamic process of a dependent variable. Therefore, to investigate the impact of financial development on renewable energy consumption, and following the practice of some literature on this topic [25,35,40], we established the dynamic panel model below:

*REC_it_* = *α* + *β*_0_*REC_it_*_–1_ + *β*_1_*FD_it_* + *γControl_it_* + *μ_i_* + *ε_i__t_* (1)

In the equation, *REC* represents renewable energy consumption; *REC_it_*_–1_ is the lag term of renewable energy consumption; *FD* denotes financial development; *Control* represents the control variables, including economic growth, industrial structure, trade openness, and foreign direct investment (FDI); *α* is the intercept term; *β*_0_, *β*_1_, and *γ* are the coefficients of lag term of renewable energy consumption, financial development, and control variables, respectively; *μ_i_* denotes the unobserved country-specific effect; *ε_i__t_* is the residual term; and *i* and *t* indicate the country and year, respectively.

We adopted the two-step generalized method of moments (GMM) [41,42,43] to estimate the regressions as it can effectively handle the issues of omitted variable biases and endogeneity, which potentially existed in dynamic panel models.

### 3.2. Data Sources

#### 3.2.1. Financial Development

As discussed above, financial development refers to the level of development of the financial sector in a certain economy or region. As an abstract concept, it can be deemed and analyzed from an overall perspective or investigated from its components, such as financial institution or market. Considering the rich connotation and complexity of financial development, we attempted to analyze its impact on renewable energy consumption from multidimensional perspectives in a unified framework, as different components of financial development may exist specific to an influential mechanism on renewable energy consumption. To achieve the above goals, we adopted an index system constructed by Svirydzenka [44] to measure financial development from various levels.

**Figure 1:**
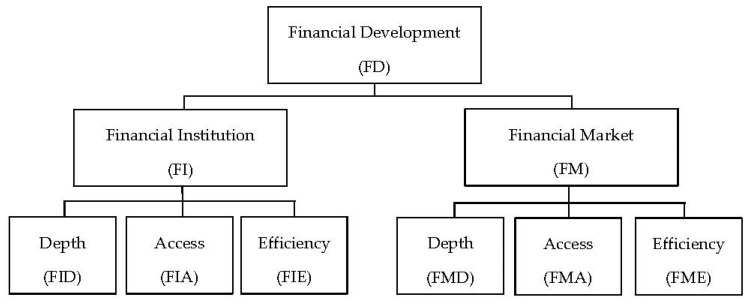
Index system of financial development constructed by Svirydzenka [44].

This index system includes nine indexes in three levels. Svirydzenka [44] divided the first-level index, the overall financial development into two second-level indexes, financial institution and financial market, which is the common practice in this research field, and each of them was measured from three third-level indexes: depth, access, and efficiency. Each of the six third-level indexes was calculated based on a series of original variables, and the upper-level indexes were calculated based on the lower-level indexes. Figure 1 presents the index system of financial development constructed by Svirydzenka [44].

The construction of this index system is based on 20 original variables, which are extracted from several mainstream financial statistics databases, such as World Bank FinStats, IMF’s Financial Access Survey, and the Bank for International Settlements (BIS) debt securities database. Each variable was carefully selected from over 100 original variables in accordance with the theories of financial development; therefore, this index system can provide a comprehensive assessment of the multiple levels of financial development. Besides, all indexes in this system were calculated with the identical methods and process, therefore enabling us to analyze the impact of financial development on renewable energy consumption in a unified framework. Several scholars adopted variables in this index system to indicate the level of financial development in empirical research [45,46,47]. The original variables, methodology, and other detailed information concerning the index system are introduced in the original literature.

To guarantee the reliability of the empirical results, we also adopted two frequently used variables, domestic credit to the private sector (% of GDP) and total value of traded stocks (% of GDP), as the proxy variables of financial development in robustness tests [48,49,50]. GDP signifies gross domestic product.

#### 3.2.2. Renewable Energy Consumption and Control Variables

Referring to the relevant empirical research of renewable energy consumption [51,52,53], we used renewable energy use (kg of oil equivalent per capita) as the proxy variable.

Based on related literature of renewable energy consumption [23,25,26,31,38] and considering data availability, we selected four control variables—economic growth, industrial structure, trade openness, and foreign direct investment (FDI)—to control the impact of other factors on the dependent variable. The details of the variables used in the empirical research are presented in Table 1.

The data contained a total sample of 103 economies covering the period from 1991 to 2014, subject to data availability. They included 28 developed economies and 75 developing economies. We further divided developing economies into emerging market economies and other developing economies for an extended empirical analysis. For simplicity, we used the word “economies” across the paper, which included countries and regions. Except the nine variables in the index system of financial development constructed by Svirydzenka [44], all other variables are selected from the WDI (World Development Indicators) database on the website of World Bank. To facilitate the explanation of empirical results and reduce non-normality and heteroscedasticity, the dependent variable REC was transformed into the form of natural logarithms.

## 4. Results and Discussions

In this section, we conducted the empirical analysis of the impact of financial development on renewable energy consumption. Section 4.1 introduced the model pretests, which confirmed the appropriateness of data selection. Section 4.2, Section 4.3 and Section 4.4 presented the empirical analysis from the three levels of financial development relying on the index system.

### 4.1. Model Pretests

Prior to empirical analysis, we conducted three pretests to verify the fitness of the data we adopted for model estimations: correlation coefficient test, panel unit root test, and panel cointegration test. The correlation coefficient test helps to distinguish the multicollinearity problem, which weakens the effectiveness of model estimation; the panel unit root test can detect nonstationary sequence data to avoid the problem of spurious regression; and the panel cointegration test verifies the existence of a cointegration relationship among variables. The results of pretests for the first regression of a full sample are displayed in Table 2, Table 3 and Table 4. The panel unit root tests included five approaches [54,55,56,57,58], and the panel cointegration tests included three approaches [59,60,61] to guarantee the reliability of results.

The results show that all regressions passed pretests that were appropriate for further empirical investigations. Specifically, for each regression, the coefficients in correlation matrices were less than 0.85, which implied that the estimation would not be significantly affected by a multicollinearity problem; the results of the unit root tests indicate that all variables were stationary sequences that did not contain a unit root; the cointegration tests confirmed that variables presented a long-term cointegration relationship.

Generally, each regression requires pretests before model estimations. However, as all these tests are used to check the fitness of data and did not provide highly correlated information concerning the research topic, we temporarily reported the results of pretests for the first regression of a full sample (Table 5, Column 2) only. All results of other regressions are available for readers upon request.

### 4.2. Analysis from First Level of Financial Development

First, we examined the impact of the overall financial development on renewable energy consumption based on the first-level index FD, and further investigated the national heterogeneity by dividing the samples into developed economies and developing economies. Table 5 presents the corresponding empirical results.

The GMM estimation requires mis-specification tests to guarantee the appropriateness of regression results, which includes the first-order and second-order serial correlation tests and the Hansen test. From Table 5, we can notice that all three regressions passed mis-specification tests according to the estimation mechanism of GMM. Specifically, the statistics of the first-order serial correlation test, AR (1), were highly significant, while the statistics of the second-order serial correlation test, AR (2), were insignificant, which implied that the results were affected by first-order autocorrelation but not affected by second-order autocorrelation. The statistics of the Hansen test were insignificant, which confirmed the appropriateness of instrumental variables in the models.

The regression result of a full sample indicated that the overall financial development had a positive impact on renewable energy consumption, which was significant at the 1% level. This practice neglected the multiple levels of financial development and the characteristics of different economies; however, the result can provide us an intuitive perception on the nexus between financial development and renewable energy consumption from the comprehensive perspective, which enriches the empirical evidence that the development of the financial sector plays an positive role in promoting the consumption of renewable energy from the macro level. Based on the quantitative data of financial development (FD), it presents a rising trend from the macro perspective in recent decades. Specifically, the average of FD for all sample countries was 0.24 in 1991, which dramatically increased to 0.38 in 2014. This further proves that financial development might be an effective implement to promote the consumption of renewable energy. This conclusion is consistent with some previous empirical research [24,25,27].

The regression results of developed and developing economies verified the existence of national heterogeneity for the influence of financial development on renewable energy consumption. Specifically, the coefficient of FD for developed economies was positive and significant at the 1% level, implying that the overall financial development could promote renewable energy consumption in developed economies, while the coefficient of FD for developing economies was also positive but insignificant, indicating that the overall financial development has no obvious effect on renewable energy consumption in developing economies.

This circumstance reflects the complexity of the influence of financial development on renewable energy consumption in different types of economies. On the one hand, although the theoretical analysis developed various influential mechanisms of financial development on renewable energy consumption, they were generally established on the ideal state, which required strict conditions. In reality, the objective conditions of different economies, such as institutional environment, technical level, and economic policies, have conspicuous disparity, which may interfere with such influential mechanisms and further lead to the discrepancy of the influence of financial development on renewable energy consumption in different types of economies. On the other hand, the development stage of financial sectors in developed and developing economies also has obvious difference [44]. For instance, developed economies generally possess well-functioning stock markets, while in many developing economies, stock markets are at the early stage of development, which only have small scales and imperfect functions. This may also lead to the distinction of the impact of financial development on renewable energy consumption between developed and developing economies. These results are consistent with several previous studies [30,31,35].

The quantitative data of financial development (FD) can intuitively reveal the discrepancy of the level of financial development between developed and developing economies: in the sample period, the average value of FD for developed and developing economies are 0.60 and 0.23, respectively. From the view of time dimension, the average value of FD for developed economies is 0.43 in 1991 and 0.64 in 2014. By comparison, the average value of FD for developing economies is 0.17 in 1991 and 0.28 in 2014. Although the value of FD for both types of economies significantly increased in recent decades, there still exists a remarkable gap in the level of financial development between developed and developing economies.

The results of national heterogeneity can, to a certain degree, explain the differential conclusions in the empirical research on the nexus between financial development and renewable energy consumption based on a diverse sample of regions.

There are 75 developing economies in our sample, although we regarded them as a whole in the above analysis. Considering the internal difference in developing economies, we further examined the potential heterogeneity by dividing them into two groups: emerging market economies and other developing economies. Table 6 presents the empirical results.

According to Table 6, we found that although the coefficient of developing economies was insignificant when they were deemed whole, further regressions detected the existence of internal heterogeneity among developing economies. Specifically, the coefficient of FD was positive and significant for emerging market economies but insignificant for other developing economies. This suggests that the overall financial development had a positive effect on renewable energy consumption in emerging market economies, although its significance level was only 10%, and had no remarkable impact in other developing economies.

Emerging market economies refer to those economies that possess a high rate of economic growth and an immense developmental potential of markets, which are quite different from developing economies in general. Actually, many scholars have taken emerging market economies as research objects due to their distinctive developmental stage [62,63,64]. The empirical results indicate that although the coefficient was not highly significant, the influential mechanism of financial development on renewable energy consumption was effective to a certain degree in emerging market economies compared with other developing economies. This further reflects national differences, which we should discreetly regard while investigating the relationship between financial development and renewable energy consumption.

### 4.3. Analysis from Second Level of Financial Development

Second, we analyzed the influence of different components of financial development on renewable energy consumption based on the second-level indexes FI and FM from the perspectives of both a full sample and subsamples. Table 7 and Table 8 display the regression results.

The empirical results reflect the relatively complex effect of a financial institution and a financial market on renewable energy consumption based on different groups of economies. For the regressions of a full sample, the coefficient of FI was significantly positive, while the coefficient of FM was insignificant, implying that a financial institution could promote renewable energy consumption, but a financial market has no obvious effect on it from the global view. For the regressions of developed economies, both coefficients of FI and FM were significantly positive, indicating that the two aspects of financial development have a positive influence on renewable energy consumption. For the regressions of developing economies, the coefficient of FI was positive and significant, while the coefficient of FM was not, signifying the distinct effect of a financial institution and a financial market on renewable energy consumption in the group of developing economies.

Financial institutions, mainly including banks, generally have a long history of development and have established a mature and wide-coverage business network in both developed and developing economies. Although there exists a discrepancy on management model and organizational structure, financial institutions possess sufficient volumes to support the development of the renewable energy industry. Besides, due to the long period of construction and operation and the requirement of large investment at the early stage, the renewable energy industry needs massive long-term funds, which can be provided by financial institutions.

The development stages of financial markets, which mainly include stock market and bond market, have remarkable disparity between different types of economies. Take the stock market as an example; stock markets in developed economies generally experience a long period of development, which possess enormous scales and a well-developed operation mechanism. They provide opportunities of direct financing for renewable-energy-related projects with a low threshold to reduce their financing costs. Besides, the strict information disclosure system of stock markets applies considerable pressure on high pollution enterprises, which forces them to employ more renewable energy. Conversely, only a part of developing economies have established stock markets, which generally have limited scales and an imperfect operation mechanism. Subject to strict access requirements, many renewable energy enterprises cannot obtain opportunities to finance stock markets.

The results of a full sample reflect the comprehensive effects of a financial institution and a financial market on renewable energy consumption. Considering the previous empirical results in Section 4.2, it might be the financial institution that dominates the positive effect of the overall financial development.

### 4.4. Analysis from Third Level of Financial Development

Third, we investigated the impact of financial development on renewable energy consumption from more concrete angles based on six third-level indexes. Table 9 presents the results of FID, FIA and FIE, while Table 10 displays the results of FMD, FMA, and FME.

The empirical results examined the effect of three aspects of a financial institution and a financial market, depth, access, and efficiency, on renewable energy consumption. According to Svirydzenka [44], depth refers to the degree of size and liquidity, access indicates the opportunities of a market subject to obtain financial services, and efficiency represents whether financial resources have been sufficiently utilized for both a financial institution and a financial market.

From Table 9, we can notice that all coefficients of three third-level indexes were positive and significant at least at the 10% level, indicating that all three aspects of a financial institution have a positive impact on renewable energy consumption. Nevertheless, the results in Table 10 signify that the coefficient of FME was significantly positive while FMD and FMA were not, implying that only the efficiency of a financial market had a positive influence, while the effects of access and depth were insignificant.

These results further confirm that the financial institution might act as the main driving force of renewable energy consumption. However, although we did not detect a significant effect of the depth and access of the financial market, the results indicate that the improvement of efficiency could promote consumption of renewable energy. Furthermore, we did not discover the existence of a negative effect of the financial market with both the second-level and third-level indexes, and the impact of the financial market in developed economies was significantly positive. All these circumstances might indicate that the influence of the financial market could promote renewable energy consumption when its development reaches a relatively high level.

Subject to data availability, the samples of FMA and FME are less than 103 economies; therefore, the results cannot be strictly compared with other regressions, which are for rough analysis only.

## 5. Robustness Tests

### 5.1. Different Variables of Financial Development

In this paper, we investigated the impact of financial development on renewable energy consumption by adopting an index system constructed by Svirydzenka [44]. As introduced above, a series of variables have also been widely used to represent the level of financial development. To guarantee the reliability of the empirical results, we selected two representative variables, domestic credit to the private sector (% of GDP) (FDR1) and total value of traded stocks (% of GDP) (FDR2), as the proxy variables to conduct the robustness tests in this section.

FDR1 contained samples of 103 economies, consistent with the previous regressions; however, FDR2 only included 60 economies due to data availability. Therefore, we conducted the regressions of a full sample and subsamples for FDR1, while only a full sample for FDR2. Table 11 and Table 12 present the results of FDR1 and FDR2, respectively.

The variable of FDR1 is widely adopted to represent the level of overall financial development. The empirical results in Table 11 show that the coefficients of FDR1 were significantly positive for the regressions of a full sample and developed economies, while insignificant for developing economies, which implies that financial development could promote renewable energy consumption in the groups of a full sample and developed economies, while it has no obvious impact in the groups of developing economies. These results are consistent with the results of regressions using the variable of FD.

The empirical results in Table 12 indicate that the coefficient of FDR2 was insignificant, suggesting that financial development has no obvious influence on renewable energy consumption when represented by FDR2. This might be because FDR2 is the variable concerning stock market, generally signifying the development of the financial market. As investigated above, the results of the financial market was insignificant for a full sample regression using the index of FM; therefore, the results of FDR2 were consistent with the above analysis that the financial market has no apparent effect on renewable energy consumption from the global view.

### 5.2. Different Variables of Renewable Energy Consumption

The commonly used variable of renewable energy consumption in empirical research is renewable energy consumption per capita. In the research of other objects, scholars also measured these objects using the ratio of their primitive values to GDP, such as carbon emissions, which is called carbon intensity, represented as CO_2_ emissions per unit of GDP. Both these two approaches enabled variables to be comparable across economies to adopt in the empirical research. However, little empirical literature has used the second approach to signify renewable energy consumption. In this section, we attempted to conduct an informal analysis on the relationship between financial development and renewable energy consumption using a new variable, RECR (ratio of primitive values of renewable energy consumption to GDP), measuring the level of renewable energy consumption. Compared with population, GDP measures the economic scale of an economy; therefore, this new variable enabled renewable energy consumption to be comparable across economies in another level, which could be used to verify the reliability of empirical results from a different perspective. As this is an informal attempt, we temporarily only examined the effect of overall financial development. All settings were similar to the regressions in Table 5, except the dependent variable. Table 13 presents the empirical results.

The results indicate that the coefficients of FD were positive with significance under the 10% and 5% levels for a full sample and developed economies, respectively. Meanwhile, the coefficient for developing economies was insignificant. Compared with Table 5, we can notice that the results were similar when renewable energy consumption was measured from the angles of per capita and per unit of GDP, as both of them represented the volume of an economy.

Nevertheless, both of the primitive values of renewable energy consumption and GDP of an economy change over time; therefore, the variable RECR lacks rigorous connotation at present. This might be why this variable is not widely adopted in empirical research. The above investigation is an informal attempt for reference only.

## 6. Conclusions and Policy Implications

In this paper, we analyzed the impact of financial development on renewable energy consumption from a multidimensional perspective based on a dynamic panel model and the panel data of 103 economies. We attached importance to the multiple levels of financial development and national heterogeneity across our research. According to the empirical results, we conclude that: (1) The overall financial development had a significantly positive influence on renewable energy consumption from the global view and in developed economies, but invalid in developing economies. Further analysis on developing economies detected a certain positive effect in emerging market economies. (2) For two main components of financial development, the financial institution was detected to have a positive impact on renewable energy consumption in all three groups, while this impact only existed in developed economies for the financial market. (3) Empirical research based on six third-level indexes of financial development showed that all three aspects, depth, access, and efficiency, of the financial institution had a positive impact on renewable energy consumption, while this effect only existed in the aspect of efficiency for the financial market. Besides, two set of robustness test further verified the reliability of the empirical results.

According to the empirical results, we suggest the following policy implications: First, the empirical analysis confirmed the positive impact of financial development on renewable energy consumption from the macro level, and did not find any evidence of negative effect across the research. Therefore, we suggest to policymakers to attach importance to this effective tool when formulating renewable-energy-related policies. Actually, the renewable energy industry generally relies on fiscal subsidies of a government at the initial stage, which are finite and unsustainable in the long term. Our research indicates that financial sectors could effectively provide support for the renewable energy industry, which facilitates its sustainable development. Second, due to the existence of a heterogenous effect between developed and developing economies, we suggest to policymakers in developing economies to emphasize the effect of financial institutions on promoting renewable energy consumption, as they could offer sufficient and stable funds that enable renewable energy enterprises to expand scales and upgrade technologies. On this basis, governments can also provide specific policy support, such as special loans and preferential interest rates, to mitigate financing constraints of renewable energy enterprises. Third, although the empirical results indicate that the effect of the overall financial market was insignificant in developing economies, the improvement of efficiency had obviously an impact on renewable energy consumption. When the development of the financial market reaches a higher level, its positive effect becomes more significant, according to the empirical analysis. Therefore, we suggest to policymakers in developing economies to consider the financial market as an important supplement to promote renewable energy consumption. On the one hand, they could improve the relevant legal and institutional arrangement of financial markets, which optimizes their function of resource allocation; on the other hand, as supplementary measures, they can reduce the access conditions of stock markets, or provide a guarantee of bond issuance for renewable energy enterprises, to further expand their financing channels.

There are several limitations of this research: (1) We have detected the discrepancy of the influence of financial development on renewable energy consumption between different types of economies, and analyzed its potential reasons; however, we are unable to further investigate this issue from an empirical perspective due to the lack of appropriate data. (2) We have adopted the panel data of 103 economies to examine the impact of financial development on renewable energy consumption; nevertheless, we did not differentiate the various types of renewable energy consumption. (3) Following general practice on this topic, we conducted our research using macrolevel data, which cannot investigate the microcosmic entity, such as enterprises and consumers, due to the lack of data set and relevant literature.

Considering the above limitations, we provide the following suggestions for future research: (1) The discrepancy of the impact of financial development on renewable energy consumption between different types of economies can be investigated in depth from both theoretical and empirical perspectives, which can help us to further clarify the finance–renewable energy nexus. (2) Scholars could focus on the analysis of the effect of financial development on specific types of renewable energy consumption, such as solar and wind energy consumption, as it can provide us a more concrete influential mechanism of financial sectors on renewable energy consumption. (3) It is necessary to research the relationship between financial development and renewable energy consumption at the micro level with the updated data set in the future or the data collected by big data technology, which can provide distinctive empirical evidence and facilitate renewable-energy-related policymaking.

## Figures and Tables

**Table 1:** Descriptions of variables.

Variable	Symbol	Indicator
Renewable energy consumption	REC	Renewable energy use (kg of oil equivalent per capita)
Overall financial development	FD	FD index constructed by Svirydzenka [44]
Financial institution	FI	FI index constructed by Svirydzenka [44]
Financial market	FM	FM index constructed by Svirydzenka [44]
Depth of financial institution	FID	FID index constructed by Svirydzenka [44]
Access of financial institution	FIA	FIA index constructed by Svirydzenka [44]
Efficiency of financial institution	FIE	FIE index constructed by Svirydzenka [44]
Depth of financial market	FMD	FMD index constructed by Svirydzenka [44]
Access of financial market	FMA	FMA index constructed by Svirydzenka [44]
Efficiency of financial market	FME	FME index constructed by Svirydzenka [44]
Financial development (for robustness test)	FDR1	Domestic credit to the private sector (% of GDP)
	FDR2	Total value of traded stocks (% of GDP)
Economic growth	EG	GDP per capita growth (annual %)
Industrial structure	IS	Industrial value added (% of GDP)
Trade openness	TO	Total import and export (% of GDP)
Foreign direct investment	FDI	Foreign direct investment (% of GDP)

**Table 2:** Correlation matrix of the variables.

Variable	REC	FD	EG	IS	TO	FDI
REC	1.0000					
FD	0.1199	1.0000				
EG	0.0222	−0.0178	1.0000			
IS	−0.2878	−0.1372	−0.0081	1.0000		
TO	−0.0518	0.0323	0.0305	0.1603	1.0000	
FDI	−0.0168	0.0624	0.1008	−0.0121	0.3001	1.0000

**Table 3:** Results of unit root tests.

Variable	LLC	HT	Breitung	IPS	Fisher
REC	−2.6543 *** (0.0040)	0.6769 ** (0.0327)	−2.4176 *** (0.0078)	−2.1709 ** (0.0150)	−9.2680 *** (0.0000)
FD	−8.2842 *** (0.0000)	0.8093 *** (0.0000)	−3.7619 *** (0.0001)	−4.6769 *** (0.0000)	−15.8584 *** (0.0000)
EG	−19.0470 *** (0.0000)	0.3699 *** (0.0000)	−3.8096 *** (0.0001)	−16.0529 *** (0.0000)	−19.8053 *** (0.0000)
IS	−12.4354 *** (0.0000)	0.9745 *** (0.0000)	−5.3547 *** (0.0000)	−3.0556 *** (0.0011)	−14.0685 *** (0.0000)
TO	−7.5101 *** (0.0000)	0.9669 *** (0.0000)	−3.5309 *** (0.0002)	−3.1396 *** (0.0008)	−13.5660 *** (0.0000)
FDI	−3.9216 *** (0.0000)	0.2461 *** (0.0000)	−4.2734 *** (0.0000)	−13.5136 *** (0.0000)	−16.3613 *** (0.0000)

Notes: LLC signifies the Levin–Lin–Chu test, HT signifies the Harris–Tzavalis test, and IPS signifies the Im–Pesaran–Shin test. Values in parentheses are the *p*-values. *** and ** indicate significance at the 1% and 5% levels, respectively.

**Table 4:** Results of cointegration tests.

Approach	Statistics
Kao	6.2155 ***(0.0000)
Pedroni	−5.8244 ***(0.0000)
Wester Lund	4.6816 ***(0.0000)

Notes: Values in parentheses are the *p*-values. *** indicates significance at the 1% level.

**Table 5:** Regression results of first level of financial development.

Variable	Full Sample	Developed Economies	Developing Economies
L.REC	0.99578 ***(71.48)	0.88121 ***(28.05)	0.99146 ***(48.76)
FD	0.05144 ***(4.02)	0.21430 ***(3.16)	0.01587(0.60)
EG	0.01734 ***(3.23)	−0.00619(−0.18)	0.02023 ***(3.43)
IS	−0.07789(−1.51)	0.42503(0.88)	−0.07658(−1.39)
TO	0.02981 ***(2.88)	0.15193 **(2.01)	0.02689 *(1.85)
FDI	0.01475(0.42)	−0.05466 *(−1.76)	0.01355(0.17)
AR (1)	−5.19 ***(0.000)	−4.21 ***(0.000)	−4.30 ***(0.000)
AR (2)	−0.59(0.554)	−0.97(0.330)	−0.12(0.908)
Hansen test	90.25(0.301)	26.48(0.151)	68.62(0.389)
No. economies	103	28	75

Note: L.REC is the first-order lag term of REC. AR (1) denotes the first-order autocorrelation estimator. AR (2) represents the second-order autocorrelation estimator. No. economies indicates the number of economies. For all variables, values in parentheses are t-statistics. For AR (1), AR (2), and the Hansen test, values in parentheses are the *p*-values. ***, **, and * indicate levels of significance at 1%, 5%, and 10%, respectively. These notes are identical in the following tables.

**Table 6:** Regression results of the first level of financial development (developing economies).

Variable	Emerging Market Economies	Other Developing Economies
L.REC	0.96129 ***(12.73)	0.96084 ***(38.74)
FD	0.40809 *(1.67)	−0.10224(−1.08)
EG	0.05492 **(2.04)	0.02002 ***(3.31)
IS	−0.16992 *(−1.79)	−0.07257(−0.85)
TO	0.02673 *(1.73)	0.02943(1.17)
FDI	−0.03113(−1.27)	−0.03874(−0.44)
AR (1)	−2.61 ***(0.009)	−3.68 ***(0.000)
AR (2)	−0.61(0.544)	0.84(0.402)
Hansen test	11.07(0.352)	45.92(0.393)
No. economies	20	55

Note: ***, **, and * indicate levels of significance at 1%, 5%, and 10%, respectively.

**Table 7:** Regression results of second level of financial development (FI).

Variable	Full Sample	Developed Economies	Developing Economies
L.REC	0.87128 ***(26.41)	0.86981 ***(28.09)	0.94395 ***(32.75)
FI	0.12905 ***(2.63)	0.29495 ***(2.73)	0.16911 **(2.21)
EG	0.03076 ***(2.78)	−0.00410(−0.15)	0.02967 ***(3.36)
IS	−0.03956 *(−1.79)	0.25962(0.38)	−0.01973(−1.44)
TO	0.01324(0.44)	0.11913(1.64)	0.01415(0.60)
FDI	−0.01150(−0.29)	−0.06451 **(−2.05)	−0.01304(−0.14)
AR (1)	−5.09 ***(0.000)	−4.19 ***(0.000)	−4.25 ***(0.000)
AR (2)	−0.78(0.434)	−0.97(0.333)	−0.36(0.718)
Hansen test	80.63(0.123)	25.08(0.570)	69.08(0.374)
No. economies	103	28	75

Note: ***, **, and * indicate levels of significance at 1%, 5%, and 10%, respectively.

**Table 8:** Regression results of second level of financial development (FM).

Variable	Full Sample	Developed Economies	Developing Economies
L.REC	0.97708 ***(80.54)	0.87438 ***(26.93)	0.99333 ***(47.13)
FM	0.02980(1.18)	0.15445 ***(3.01)	0.01242(0.74)
EG	0.01865 ***(3.04)	−0.01206(−0.40)	0.02367 ***(3.60)
IS	−0.01629(−0.73)	0.50749(0.90)	−0.07438(−1.10)
TO	0.04012 ***(4.00)	0.16301 **(2.26)	0.03079 ***(2.71)
FDI	−0.01768(−0.74)	−0.06511 **(−1.96)	0.00172(0.02)
AR (1)	−5.23 ***(0.000)	−4.21 ***(0.000)	−4.26 ***(0.000)
AR (2)	−0.77(0.443)	−0.97(0.332)	−0.35(0.726)
Hansen test	91.20(0.330)	26.21(0.159)	67.90(0.412)
No. economies	103	28	75

Note: *** and ** indicate levels of significance at 1% and 5%, respectively.

**Table 9:** Regression results of third level of financial development (FID, FIA, and FIE).

Variable	FID	FIA	FIE
L.REC	0.99526 ***(67.75)	0.99381 ***(71.06)	0.87714 ***(28.28)
FID/FIA/FIE	0.03768 **(2.38)	0.04956 ***(4.70)	0.16311 *(1.81)
EG	0.01978 ***(3.40)	0.01938 ***(3.28)	0.02021 *(1.94)
IS	−0.07960(−1.55)	−0.07686(−1.53)	−0.04446(−1.56)
TO	0.03612 ***(4.01)	0.03286 ***(3.71)	0.02967 ***(3.75)
FDI	0.00554(0.17)	0.00374(0.11)	0.00970(0.27)
AR (1)	−5.21 ***(0.000)	−5.21 ***(0.000)	−5.13 ***(0.000)
AR (2)	−0.79(0.429)	−0.80(0.427)	−0.76(0.445)
Hansen test	93.65(0.221)	92.05(0.257)	87.04(0.303)
No. economies	103	103	103

Note: ***, **, and * indicate levels of significance at 1%, 5%, and 10%, respectively.

**Table 10:** Regression results of third level of financial development (FMD, FMA, and FME).

Variable	FMD	FMA	FME
L.REC	0.84180 ***(18.09)	0.84662 ***(19.41)	0.84764 ***(25.54)
FMD/FMA/FME	0.08029(1.21)	0.08592(1.43)	0.22823 ***(4.39)
EG	0.02666 **(1.97)	0.04144 ***(2.62)	0.03322 **(2.03)
IS	−0.05398 **(−2.09)	−0.08710 ***(−2.77)	−0.09208 **(−2.39)
TO	0.03687(0.94)	0.02866(0.71)	0.01542(0.40)
FDI	−0.00586(−0.13)	−0.01650(−0.41)	−0.08725(−1.51)
AR (1)	−5.00 ***(0.000)	−5.47 ***(0.000)	−4.77 ***(0.000)
AR (2)	−0.72(0.471)	−0.73(0.463)	−0.79(0.429)
Hansen test	81.14(0.115)	77.88(0.171)	74.28(0.253)
No. economies	103	88	84

Note: *** and ** indicate levels of significance at 1% and 5%, respectively.

**Table 11:** Regression results with FDR1.

Variable	Full Sample	Developed Economies	Developing Economies
L.REC	0.99596 ***(68.85)	0.75648 ***(15.66)	0.98688 ***(42.56)
FDR1	0.02382 ***(3.10)	0.23314 **(2.04)	−0.00317(−0.34)
EG	0.01795 ***(3.29)	−0.02324(−0.60)	0.02106 ***(3.46)
IS	−0.07008(−1.40)	0.91519(0.66)	−0.08526(−1.44)
TO	0.02790 **(2.52)	0.08305(0.84)	0.02643 *(1.74)
FDI	0.01573(0.42)	−0.11230 **(−2.07)	0.01412(0.18)
AR (1)	−5.19 ***(0.000)	−3.89 ***(0.000)	−4.30 ***(0.000)
AR (2)	−0.60(0.548)	−0.96(0.337)	−0.12(0.906)
Hansen test	91.51(0.270)	23.10(0.187)	68.79(0.383)
No. economies	103	28	75

Note: ***, **, and * indicate levels of significance at 1%, 5%, and 10%, respectively.

**Table 12:** Regression results with FDR2.

Variable	Full Sample
L.REC	0.77194 ***(17.26)
FDR2	0.04773(1.31)
EG	−0.01871(−0.78)
IS	−0.05623(−0.15)
TO	0.01587(0.23)
FDI	−0.04109(−0.98)
AR (1)	−4.49 ***(0.000)
AR (2)	−0.37(0.715)
Hansen test	48.92(0.282)
No. economies	60

Note: *** indicates level of significance at 1%.

**Table 13:** Regression results with RECR.

Variable	Full Sample	Developed Economies	Developing Economies
L.RECR	0.92831 ***(28.20)	0.82761 ***(16.88)	0.94263 ***(17.34)
FD	0.23070 *(1.71)	0.21318 **(2.17)	−0.24189(−0.97)
EG	−0.01391 ***(−4.99)	−0.09059 **(−2.47)	−0.09732 ***(−5.31)
IS	−0.03361(−1.61)	0.40974(0.68)	−0.02912(−1.36)
TO	−0.01370(−0.31)	0.15031 *(1.70)	−0.02092(−0.05)
FDI	−0.02334(−0.39)	−0.08176 *(−1.83)	0.01555(0.12)
AR (1)	−5.07 ***(0.000)	−4.11 ***(0.000)	−4.10 ***(0.000)
AR (2)	−0.50(0.618)	−0.94(0.346)	−0.12(0.904)
Hansen test	96.19(0.283)	25.89(0.257)	55.33(0.118)
No. economies	103	28	75

Note: ***, **, and * indicate levels of significance at 1%, 5%, and 10%, respectively.

## Data Availability

The sources of all data used for the analysis are provided in the main text.
